# Is the routine use of a water-soluble contrast enema prior to closure of a loop ileostomy necessary? A review of a single institution experience

**DOI:** 10.1186/s12957-015-0742-z

**Published:** 2015-12-04

**Authors:** Nikoletta Dimitriou, Sofoklis Panteleimonitis, Ajit Dhillon, Kirsten Boyle, Mike Norwood, David Hemingway, Justin Yeung, Andrew Miller

**Affiliations:** Colorectal Department, Leicester Royal Infirmary, Leicester, UK; 26, Mikras Asias str, Athens, 11527 Greece

**Keywords:** Anastomotic leakage, Water-soluble contrast enema, Colorectal cancer

## Abstract

**Background:**

The aims of the study were to determine the radiological leak rate in those patients who had undergone a resection for left-sided colorectal cancer and to see if the presence of a leak can be related with the postoperative clinical period. We also aimed to identify any common factors between patients with leak.

**Methods:**

A retrospective analysis of prospectively collected data of all patients who underwent a left-sided colorectal cancer resection with formation of a defunctioning ileostomy was undertaken. Between 2005 and 2010, 418 such patients were identified.

**Results:**

A water-soluble contrast enema was performed in 339 patients (81.1 %). Of these, 24 (7.1 %) were reported to show an anastomotic leak. Data for these 24 patients is presented in this study.

Twenty-three (95.8 %) of the leaks occurred in patients who had undergone an anterior resection; 95.8 % of the patients with a leak were male. Fifteen (62.5 %) patients underwent neo-adjuvant radiation. The mean length of stay in those patients shown to have a subsequent radiological leak was 18.8 days (median), compared with the overall unit figures of 12 days. Only 29.2 % of the patients who had a leak identified had an uncomplicated postoperative period. Overall 87.5 % of the patients had a reversal of the ileostomy.

**Conclusions:**

Radiological leakage is not uncommon. The majority of patients, who were shown to have a radiological leak in this study, were male, had undergone an anterior resection, had received neo-adjuvant radiation, had a longer initial length of stay and had postoperative complications. Water-soluble contrast enemas could be selectively used in patients with these characteristics.

## Background

Anastomotic failure following a colorectal resection is a devastating complication for both the patient and surgeon. It is associated with increased morbidity and mortality [1, 2]. Mortality rates are reported to be between 6 and 22 % [3]. The Association of Coloproctology of Great Britain and Ireland Guidelines recommend that the audited leak rate for anterior resections should be below 8 % and below 4 % for all other resections [4]. The literature reports anastomotic leakage to be between 11 and 12 % after rectal surgery [5, 6] compared with 3–4 % after colonic surgery [7].

The aim of forming a defunctioning loop ileostomy is to divert the faecal stream from the anastomosis. The role of diverting the faecal stream from the anastomosis is still controversial. In a recent meta-analysis of four randomized controlled trials, Huser et al. showed a statistically significant increase in clinical anastomotic leakage and the need for reoperation in patients without a proximal stoma after low anterior resection for cancer [8]. The same results were reported in a Cochrane review [9]. Others have argued that the stoma mitigates the consequences of a leakage but does not lower the leakage rate itself, as was the result in a large retrospective multicenter study by Gastinger et al., in which leakage rate was 14 % with and without a defunctioning stoma [10]. A more recent randomized multicentre study concluded that the presence of a defunctioning stoma significantly decreases the rate of symptomatic leakage [11]. A review of almost 2000 patients reported that a defunctioning stoma following a coloanal anastomosis appears to protect against postoperative sepsis, septic shock and need for reoperation; it is overused in patients having a more proximal anastomosis and should be avoided in low-risk patients [12].

Defunctioning loop ileostomies are usually reversed 8–12 weeks from initial surgery [13]. If however, adjuvant chemotherapy is indicated, the time to reversal may be somewhere in the order of 35–40 weeks. In many centres, a water-soluble contrast enema (WSCE) is routinely performed to assess the integrity of the colorectal anastomosis prior to closure. However, such routine practise is controversial. Kalady et al. have shown that a routine WSCE did not reveal any anastomotic leaks that were not already suspected clinically [14]. Jayaraja et al. have also shown that in cases where colonic pouches are formed, the appearances of WSCE can be mistaken for leaks leading to false positive results [15].

The primary aim of our study was to determine the radiological leak rate in those patients who had undergone a resection for left-sided colorectal cancer and to see if the presence of a leak relates with postoperative complications. We also aimed to identify any characteristics in the patients who were identified to have a positive radiological leak. This could help us identify a group of patients who could selectively have a WSCE prior to ileostomy closure.

## Methods

A prospective database of all patients with a diagnosis of colorectal cancer has been maintained since 1998 in Leicester. This database was interrogated. A retrospective analysis of all patients who underwent surgery for colorectal cancer with formation of a defunctioning ileostomy over a period of 6 years was performed. Between 2005 and 2010, 418 such patients were identified.

Using our intranet radiology reporting system (IMPAX, AGFA Healthcare N.V., Mortsel, Belgium), we reviewed all 418 patients’ radiology records and identified those who had a WSCE following their resection. All WSCE were reported by a consultant GI radiologist. If the results were in doubt, the images were discussed together with the colorectal team consultants. We reviewed all of the medical notes of those patients with a positive radiological leak, in order to identify any postoperative complications. Data was recorded using a pro forma specifically designed for this study.

## Results

Four hundred eighteen patients underwent resectional surgery with formation of a defunctioning loop ileostomy. Of these, 339 (81.1 %) had a WSCE. Out of these 339, 24 (7.1 %) were reported to show an anastomotic leak. Twenty-three (95.8 %) of the leaks occurred in patients who had undergone an anterior resection, of which there were 307. This equates to an overall leak rate of 7.5 % in the anterior resection group.

In the leakage group, 23 patients were male and the median age was 64 years. We obtained the notes for all 24 patients.

We present the data retrieved from the medical records of these 24 patients. Patient characteristics and clinical outcome are presented in Table 1.

**Table 1:** Patients characteristics and clinical outcome

	Gender	Age	Cancer site	Comorbidities	BMI	Smoking	Alcohol U/week	ASA	Neo-adjuvant therapy	Complications	Length of hospital stay
1	Male	73	Sigmoid	HTN, CHD, CKD Stage 4	29	N	15	3	N	–	18
2	Male	55	Rectum	–	31	N	22	2	Y	Pelvic collection on CT	40
3	Male	50	Rectum	–	32	N	4	2	Y	Pelvic collection, on CT	10
4	Male	62	Rectum	HTN	29	Ex smoker	28	2	Y	–	14
5	Male	66	Rectum	NIDDM, CHD, HTN	31	Ex smoker	–	3	Y	Ileus	15
6	Male	68	Rectum	–	25	Ex smoker	28	2	Only radiation	Wound dehiscence, pelvic collections-EUA, wash out	38
7	Male	77	Rectum	CHD	25	No	2	3	Y	Pelvic collection on CT, AF	23
8	Male	72	Rectum	–	29	Ex smoker	1	2	N	–	9
9	Male	75	Rectum	–	24	Ex smoker	–	2	N	–	12
10	Male	66	Rectosigmoid junction	–	24	N	NR	2	N	–	9
11	Male	52	Rectum	–	23	Y	2	2	Y	Pneumonia	14
12	Male	62	Rectum	–	28	Ex smoker	30	2	N	Pneumonia, ileus	27
13	Female	65	Sigmoid	–	23	N	1.5	2	N	Obstructed stoma	25
14	Male	46	Rectum	–	33	N	1	2	N	Pelvic collection-EUA, washout	8
15	Male	62	Rectum	–	24	Y	20	2	Only radiation	Pelvic collection-EUA, washout	47
16	Male	81	Rectum	COPD	32	Ex smoker	6	3	N	Pneumonia	18
17	Male	49	Rectum	HTN, CHD	34	Ex smoker	20	2	Only radiation	Pelvic collection-EUA, washout	12
18	Male	59	Rectum	–	27	Ex smoker	3	2	Y	–	5
19	Male	70	Rectum	HTN	33	N	12	2	N	Pelvic collection on CT	44
20	Male	80	Rectum	CKD STAGE 3	24	N	1	3	Only radiation	Pneumonia, AF	21
21	Male	59	Rectum	HTN	28	Y	35	2	Only radiation	AF	9
22	Male	65	Rectum	CHD, COPD	30	Ex smoker	15	2	Only radiation	Pelvic collection—USS-guided drainage	12
23	Male	54	Rectum	–	33	N	32	2	Y	–	5
24	Male	68	Sigmoid	HTN, MI	28	N	10	3	Only radiation	UTI, ileus	16

*HTN* hypertension, *NIDDM* noninsulin-dependent diabetes mellitus, *AF* artial fibrillation, *CKD* chronic kidney disease, *UTI* urinary tract infection, *CHD* coronary heart disease, *COPD* chronic obstructive pulmonary disease, *NR* not reported, *EUA* examination under anaesthesia, *Y* yes, *N* no, *U* units, *USS* ultrasound

Twenty patients had a rectal tumour, three a sigmoid tumour and one a rectosigmoid tumour. The tumour location varied from 5 to 20 cm from the anal verge.

Medical case note review revealed that 5 patients were on aspirin and 1 patient was on steroids preoperatively. Three patients were current smokers; 10 were ex-smokers. Only 6 patients exceeded the recommended weekly alcohol consumption limit (>21 units). Six patients had an ASA score of 3, and the rest of the group had an ASA score of 2. BMI range was between 23 to 34, 8 patients had a BMI between 20–25, 8 between 26–30 and 8 between 31–35. All patients had a normal albumin preoperatively, but 78 % had a low albumin postoperatively.

In total, 15 of the patients (62.5 %) received neo-adjuvant treatment. Eight of those had combined neo-adjuvant chemoradiation, and 7 had only neo-adjuvant radiotherapy. Eight patients subsequently received adjuvant chemotherapy, and 3 received adjuvant chemoradiation. Neo-adjuvant treatment consists of short course radiotherapy (25 Gy) for T2/T3a, N0 rectal cancer and neo-adjuvant chemoradiotherapy with capecitabine, oxaliplatin or 5 fluorouracil, and the radiotherapy dosage is 45–50.4 Gy for locally advanced rectal cancer.

The mean length of stay in those patients showed to have a subsequent radiological leak was 18.8 days. This exceeded the overall unit figure of 12 days. Looking at this in more detail, 23 out of 24 patients stayed over 1 week, 12 out of 24 patients were still an inpatient after 2 weeks and 4 out of 20 patients were still an inpatient after 1 month. An enhanced recovery programme was introduced universally to the colorectal unit in 2010.

Only seven (29.2 %) of these patients had no immediate postoperative complications. Nine (37.5 %) patients developed a postoperative collection or leak, confirmed by CT scan. Two of these patients required intensive treatment unit admission. Patients with collection had a drainage either radiologically or transanaly in theatre. Four patients developed postoperative ileus that resolved with conservative therapy, and two patients developed AF. Mean time from ileostomy formation to WSCE was 93.6 days.

Following identification of a positive radiological leak on WSCE, 17 patients underwent a subsequent WSCE; however, 7 did not have a subsequent WSCE. Of the 17 who had a repeat WSCE, 13 had further repeat WSCEs until no leak was identified and thereafter underwent ileostomy reversal. Three patients were found to have a positive radiological leak on their last WSCE but had their ileostomy reversed, more than 12 months from their original surgery. One of those patients subsequently found to have a local recurrence and required further surgical treatment.

Of the seven who did not have repeat WSCEs, five had their ileostomy reversed uneventfully, 1 patient was found to have liver metastases and did not wish to have his ileostomy reversed and 1 developed chronic pelvic sepsis and did not have reversal of his ileostomy.

**Fig. 1:**
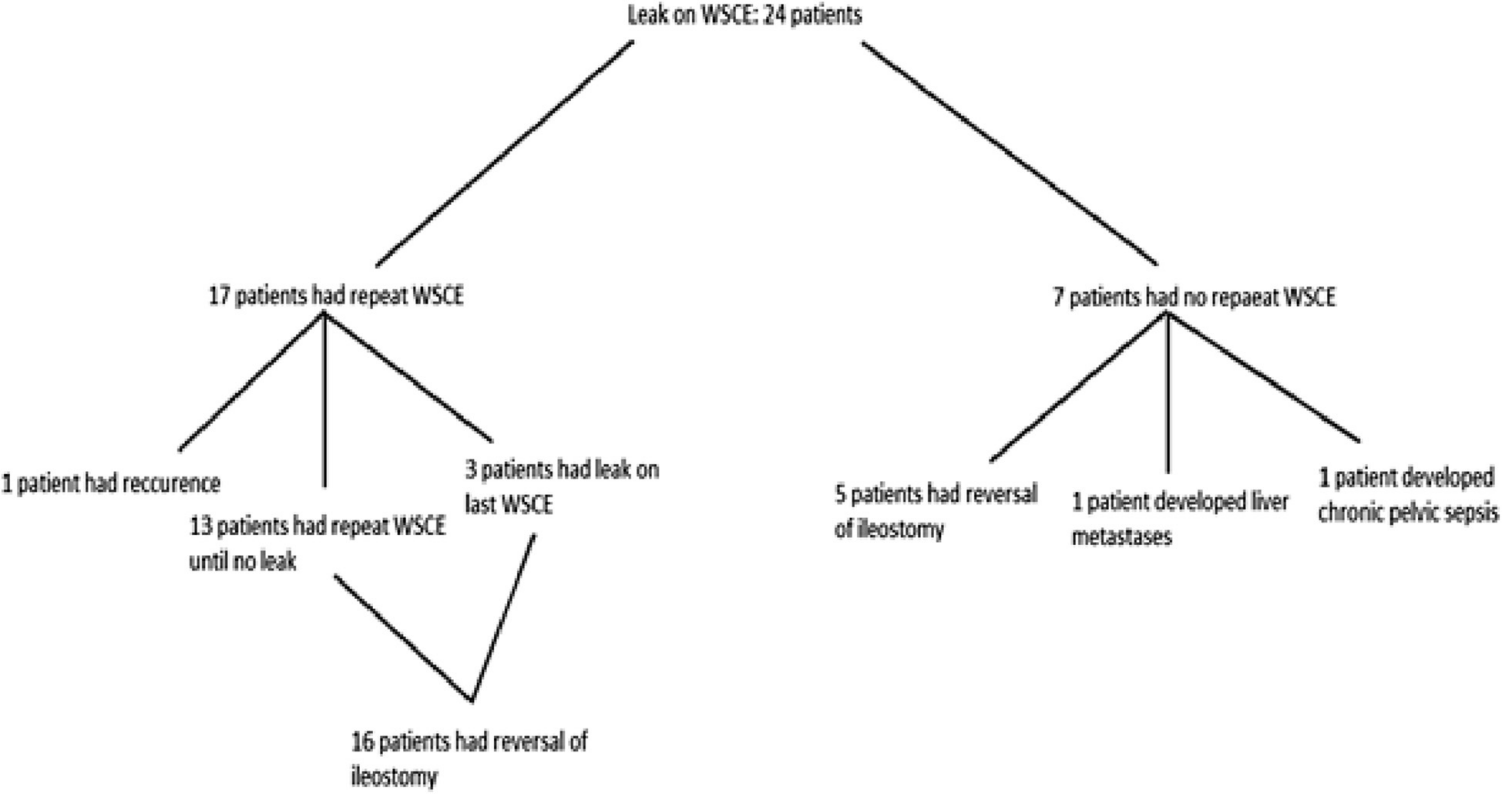
In the above diagram, the clinical course of the 24 patients with anastomotic leak is presented

However, of greater interest, seven patients underwent ileostomy closure despite their last WSCE being positive for a radiological leak. All seven patients made an uneventful recovery, but one developed recurrence at the anastomosis site. In Fig. 1, the clinical course of the 24 patients with anastomotic leak is presented.

In summary the leak rate discovered in WSCE was 7.1 %. Twenty-three out of 24 patients had an anterior resection, and 95.8 % of the patients were male. Of these patients, 62.5 % had neo-adjuvant radiotherapy. Only 29.2 % of the patients had an uncomplicated postoperative period. The mean hospital stay was 18.8 days, higher than the overall hospital stay of the unit. Finally, 87.5 % of the patients had a reversal of the ileostomy.

## Discussion

A defunctioning stoma is frequently created to minimize the impact of any subsequent anastomotic leak after a low rectal anastomosis [16].

Water-soluble contrast enemas (WSCEs) are used to assess the integrity of the anastomosis before the loop ileostomies are reversed and normal faecal stream resumes. But the routine use of WSCE is still controversial; studies have shown that WSCE fails to provide any additional information that result in a change to patient management [14, 17].

Our overall radiological leak rate was 7.1 %. The majority of patients who found to have radiological leaks had common characteristics. An anterior resection was the operation performed in 95.8 % patients with a radiological leak. In the group as a whole, 80 % of patients underwent an anterior resection. Moreover, 95.8 % of the patients were male. Of the patients who had a radiological leak, 62.5 % had neo-adjuvant radiation and 45.8 % had adjuvant chemotherapy. Our findings agree with a recent systematic review and meta-analysis that showed that anterior resection, male gender and neo-adjuvant radiotherapy are risk factors for anastomotic leakage [18].

Furthermore, it was found that the length of stay of the patients who had a radiological leak was higher than the overall unit figures (mean 18.8 vs 12). Only 29.2 % of the patients with leak had an uncomplicated postoperative period. Of the patients, 37.5 % had clinical leak confirm postoperatively with CT scan. Our data confirms a previous study that reports that all anastomotic leaks were diagnosed clinically and WSCE did not provide any additional information [19].

Thirteen (54.2 %) of our patients who had positive radiological leaks had repeat WSCEs until they had a negative WSCE before reversing the loop ileostomy. Seven patients (33.3 %) who had their ileostomies reversed whilst their last WSCE showed a radiological leak. All of these seven patients were more than 12 months from ileostomy formation and all made an uneventful recovery, but one went on to develop an anastomotic recurrence. This could indicate two things: either the time frame from the last WSCE to reversal was enough to allow the leaks to resolve or if the patients are clinically well and anastomotic leak on WSCE is not a contraindication for reversal.

In a previous study of 174 patients, 6.4 % of WSCEs showed pathology but had normal digital rectal examinations [20]. These patients had their ileostomies reversed with no subsequent complications. It is not surprising that they have suggested digital rectal examination as a more reliable alternative to WSCE. However, this is solely dependent on the experience of the performing surgeon. In another similar study, digital rectal examination and rigid proctoscopy were shown to have equal sensitivity as WSCE (100 %) [17]. The authors suggest that routine digital rectal examination and rigid proctoscopy can be used to evaluate low pelvic anastomosis, and WSCE should be used only on selective cases with abnormal findings [17].

## Conclusions

Overall, radiological leakage is uncommon. An anastomotic leak is usually clinically recognized and should always be considered in any patient who is unwell in the postoperative period. However, the majority of patients who do have a leak have undergone anterior resections and are male, have neo-adjuvant radiation and have a longer initial length of stay and have postoperative complications, either a cardiac event, fever, or post operative ileus. These characteristics could form the basis of a selective policy for the use of WSCE.
